# Emotional abuse among married women at Vhembe district: Experiences and consequences

**DOI:** 10.4102/hsag.v27i0.2002

**Published:** 2022-11-23

**Authors:** Rabelani Mulaudzi, Enneth T. Nkhwashu, Hilda N. Shilubane

**Affiliations:** 1 Department of Advanced Nursing Science, Faculty of Health Sciences, University of Venda, Thohoyandou, South Africa; 2 Private, Johannesburg, South Africa

**Keywords:** emotional abuse, women, experiences, consequences, qualitative analysis

## Abstract

**Background:**

Emotional abuse is seen as an issue to be kept secret and not reported, because there is no evidence of abuse until stress, sickness and death occur.

**Aim:**

The study aimed at exploring the experiences and consequences of emotional abuse among married women in a selected village in the Vhembe district in Limpopo province.

**Setting:**

Vhembe district in Limpopo province.

**Methods:**

This qualitative study adopted a descriptive phenomenological design. Participants were selected using purposive sampling, with the support of the local healthcare professional to select participants with lived experiences around emotional abuse. In-depth one-on-one unstructured interviews were conducted. Only ten married women were interviewed because of data saturation. Colaizzi’s method was used to analyse the data.

**Results:**

The results revealed three themes – the participants’ experiences of emotional abuse, the impacts of emotional abuse on the victim and reasons for remaining in an abusive marriage – and eight sub-themes. Married women also stated several reasons for remaining in abusive marriages such as poverty and love for the spouse.

**Conclusion:**

The study showed a lack of understanding and consequences of emotional abuse among married women in a village in Limpopo province. The cultural institutions that support *lobola* disempower women, reinforce violence and allow husbands to have power over their wives. The participants thought men had every right to do whatever they liked with them because they paid their bridal price (*lobola*). Hence, it is essential to increase the understanding of emotional abuse among women by educating them on various platforms regarding their rights.

**Contribution:**

This study contributes to understanding the lived experiences of emotional abuse and its consequences on married women.

## Introduction

Gender-based violence (GBV) is a significant public health problem. It is an abuse of constitutional rights and threatens the rights and dignity of women worldwide. Although GBV has been a theme of investigation since the 20th century, there is no comprehensive data in economically developing countries. Violence against married women is acknowledged as a major threat to human rights and a significant threat to women’s health and well-being. A study conducted by the World Health Organization found that 15% – 71% of married women experienced violence (Perrin et al. 2019; Sapkota et al. 2016).

In GBV, men are often the culprits and women the sufferers (Sapkota et al. 2016; South African Government 2021b). Violence against women is a multifaceted, widespread health challenge and a substantial contributor to morbidity and mortality among women worldwide (Christian Aid Ireland Learning Paper 2018; Mittal & Singh 2020; Muluneh et al. 2020). In support of the above statement, the World Bank (2019) indicated that 35% of women worldwide have experienced emotional abuse, which is one form of GBV.

Gender-based violence includes domestic violence, where women are abused in their homes. Domestic violence includes violence committed by a partner or family members and is demonstrated through emotional, physical and financial abuse (Mittal & Singh 2020). Tracy (2021) defines emotional abuse as any behaviour including insults, humiliation, intimidation or any other treatment that the male perpetrator uses to instil fear among married women to control them. The rise in domestic violence and domestic homicide (Bradbury-Jones & Isham 2020; Smith 2020; Wanger 2020) is a cause for concern and affects women psychologically, as they display signs and symptoms of stress. In some instances, women do not control their finances; they work very hard to earn a salary, but their money is taken away by their partners, making women feel weak and powerless with a feeling that their rights are being violated.

Most married women in African countries do not view verbal abuse and forced sexual intercourse by their husbands as a crime they can report; they believe they have no choice and must submit to their husbands as they have paid *lobola* (Auteri 2019; Muche, Adekunle & Arowojolu 2017). This is evident in this study, as participants were seen as ugly and not being able to be loved but did not report their partners. Longitudinal studies conducted in South Africa found GBV to be correlated with comprising health consequences which support the current findings (Abrahams et al. 2017; Fields et al. 2018).

A study cohort analysis of young women in rural South Africa showed that depression was associated with emotional GBV (Li, Carracher & Bird 2020). Similarly, Tsai et al. (2016) analysed data from a cohort of 1238 pregnant women in Cape Town and found that intimate partner violence (IPV) was significantly associated with depression symptoms. Tsai et al. (2016) further demonstrated that although the rates of GBV in South Africa are among the highest in the world, significant gaps exist in the literature on the links between GBV and mental health, hence the current study.

Furthermore, Kimuna, Tenkorang and Djamba (2018) and the National Gender-based Violence and Femicide Strategic Plan 2020–2030 (South African Government 2019) established that the prevention of abuse of women requires urgent attention. This necessitated the launch of the private sector Gender-Based Violence and Femicide (GBVF) Response Fund Phase 1 by President Ramaphosa in February 2021 (South African Government 2021a). The fund was created to fulfil the resolution taken during the 2018 GBVF summit to end violence against women and children.

Every culture has traditions about the significance of the home and its safety. However, for several women, the home is a place of sorrow and embarrassment. Many women are taught from a young age to be submissive, subordinate and obedient to their male counterparts, and they are less valued than men. This level of consciousness which reinforces cultural norms and expectations ensures the continuous cycle of male patriarchy (Mshweshwe 2020).

Mshweshwe (2020) further revealed that these men also prohibit their wives from talking to other men. The co-existence of threats, verbal abuse, yelling and control over economic resources reminds the victim of the possibility of physical abuse and undermines her independence (Mshweshwe 2020). Similarly, women’s roles of being vulnerable, helpless and reliant on men are supported by expectations placed on women to be obedient, child-rearing and passionate (Mshweshwe 2020). Two studies on GBV were conducted in Limpopo province, but neither of them focused on emotional abuse and its consequences on health outcomes in Limpopo province in rural areas. It was unclear how married women experienced emotional abuse in a selected village in the Limpopo province. The current study thus aimed to understand the lived experiences and consequences of emotional abuse among married women in a selected village in the Vhembe district of the Limpopo province.

## Materials and methods

### Study design

A qualitative descriptive phenomenological design was chosen to explore the experiences and consequences of emotional abuse among married women in the Vhembe district. Neubauer, Witkop and Varpio (2019) define phenomenology as a study of lived experiences. The use of a phenomenological research design was deemed appropriate to understand the meanings of the experiences lived by married women in the selected village.

### Study setting

The study was conducted at a selected village in the Vhembe district in Limpopo province. The village was selected based on the high statistics of GBV reported by the key informants at the selected village. They could not, however, supply the researcher with statistics to prove it. Vhembe is located in the northern part of Limpopo province and shares borders with the Capricorn and Mopani districts in the eastern and western directions, Zimbabwe in the north, Mozambique through the Kruger National Park in the east and Botswana in the north-west (Vhembe 2021). The district has a total population of 1 402 779 people. Fifty-four per cent of the total population are female and 46% are male. Around 56.2% are below the lower poverty line of R840 per person per month (CoGTA 2020).

### Population and sampling

The target population was women from a selected village. The purposive sampling technique was used as the participants, who were regarded as experts, could provide valuable information in the field under study (De Vos et al. 2011; Grove & Gray 2018). The inclusion criteria were married women of 18 years and older, in an abusive relationship and residing in the selected village. These women were selected with the support of a professional nurse from the local clinic. All sampled women were provided with an information sheet before being invited to participate in the study. A total of 10 women were individually interviewed for between 45 and 60 min. The sample size was determined by the saturation of information, which is when data began to repeat itself (Saunders et al. 2018; Creswell & Poth 2018).

### Data collection

Data collection was conducted using in-depth face-to-face unstructured interviews with each participant. The interview was guided by the following question: ‘share with me how emotional abuse in your marriage is for you.’ Probing questions followed, dependent on the participants’ responses. These questions were developed by the first author and piloted with one woman with similar characteristics to the participants. The questions were rephrased to be clearer before they were used in the main study, and the data of the pilot were not included in the data analysis. A digital recorder was used, with the participant’s permission, to capture and preserve the data as accurately as possible (Creswell 2013; Grove et al. 2015). Field notes were taken during the interviews. Interviews were conducted over 1 month in the participants’ homes, which they preferred and chose. The researcher, on the day of data collection, introduced the topic to the participants and put them at ease. The interviews were conducted using the participants’ languages, which are Xitsonga and Tshivenda. The interviews ranged from 45 min to 60 min per session.

### Data analysis

Before the analysis, the digitally recorded interviews were transcribed and translated from the two local languages (Tshivenda and Xitsonga) to English by a language specialist fluent in the three languages. The 10 transcripts were back-translated into the two local languages to ensure accuracy. The six Tshivenda transcripts and their English versions were checked for accuracy and meaning by the first author, while the Xitsonga transcripts and their English versions were checked by the second author. Data were analysed using Colaizzi’s method (Morrow, Rodrigue & King 2015). The researcher got to know the information by perusing all the member accounts a few times and distinguished all explanations in the records that are of direct significance to the encounters and outcomes of psychological mistreatment. Following careful consideration of the significant statements, the researcher identifies meanings relevant to the phenomenon. The researcher groups the identified meanings into themes that are shared by all accounts. The specialist consolidated the comprehensive depiction down to a short, broader explanation that caught those viewpoints considered to be crucial for the construction of the peculiarity. The scientist returned the central design articulation to all members to find out if it captured their encounters (Heydarikhayat et al. 2022). Three themes and five sub-themes emerged from the data. An independent coder verified the results, and grammar was not changed to retain direct quotations.

### Trustworthiness

Reliability was guaranteed by the four standards of validity, adaptability, consistency and confirmability (Denzin & Lincoln 2011). Trust was laid out with the members by visiting them before the meeting to establish rapport and acquaint them with the process. To guarantee creditability, the researchers permitted every member to verbally affirm that the record of her meeting was a genuine impression of what she said. The researchers relied upon field notes and an audio-recorder while transcribing information to guarantee that no data were missed.

### Ethical considerations

The University of Venda Ethics Committee approved the study (project number SHS/16/PDC/17/0408). Authorisation to conduct the research was acquired from the chief of the selected village. Participants were asked to give their informed written consent. The researcher explained the study and process to participants in a private room. They gave voluntary consent before being interviewed, and they could withdraw without any penalty, and their data would be destroyed. Only the researcher, supervisor and independent coder had access to the data. Participants were informed that information would be made available in a report without exposing their names. They were informed that counselling services were available and pre-arranged after the interviews at no cost should they need them; however, the use of the counselling services was not needed. All data were kept on a computer on the cloud, password-protected, and will be deleted 5 years after the report is published. No transcripts will reveal their names, and participants will be numbered.

## Findings

### Demographic characteristics of participants

Table 1 illustrates the demographic data of participants included in the one-on-one interviews, including their age, spoken language, employment status and educational status.

**TABLE 1:** Demographic data.

Participant number	Age	Spoken language	Employment status	Education status
01	63	Venda	Unemployed	Did not go to school
02	29	Venda	Unemployed	Grade 12
03	41	Xitsonga	Employed	Higher Education Certificate
04	48	Venda	Employed	Higher Education Certificate
05	43	Xitsonga	Employed	Grade 12
06	38	Xitsonga	Employed	Higher Education Certificate
07	32	Venda	Employed	Grade 12
08	49	Xitsonga	Unemployed	Primary education
09	35	Venda	Employed	Grade 12
10	37	Venda	Employed	Grade 12

## Themes and sub-themes

Three themes and eight sub-themes emerged from the data analysis, as shown in Table 2. The themes and sub-themes identified followed the participants’ accounts to support their experiences of emotional abuse and its impact; the appropriate quotes drawn from the data collected are contextualised with the relevant literature to substantiate the themes and sub-themes below.

**TABLE 2:** Themes and sub-themes.

Themes	Sub-themes
1. Participants’ experience of emotional abuse	1.1 Humiliation and degradation 1.2 Controlling behaviour of male partners 1.3 Withholding of affection and denial of conjugal rights
2. Impacts of emotional abuse on the victim	2.1 Feeling of hopelessness and low self-confidence 2.2 Poor mental health
3. Reasons for remaining in an abusive marriage	3.1 Commitment made before God 3.2 Poverty 3.3 Love for the person

### Theme 1: Participants’ experiences of emotional abuse

Study findings revealed that married women are often emotionally abused by their partners within their homes. Despite a well-founded and applied knowledge of the occurrence of GBV in developed countries, sexual assault and emotional abuse are still not considered criminal acts in many countries. Gender-based violence, which is legitimately denounced in many places, is still witnessed by a larger number of people. Regulations prohibiting GBV in sub-Saharan African countries are inadequate, and where they do exist, justice for perpetrators is rarely served (Okolie 2019). Three sub-themes emerged from this theme during data analysis. The following are sub-themes that emerged.

#### Sub-theme 1.1: Humiliation and degradation

The findings of the study revealed that married women are emotionally abused in similar ways in their homes. Participants mentioned specific actions enacted by their partners which are emotionally abusive. These actions include humiliation, verbal and physical abuse.

A participant was seen as ugly and not being able to be loved:

‘He usually tells me that I am ugly, and it got worse because the other day it got physical, because as he was saying, “you are ugly, you are ugly,” he took me to a mirror and said, “take a good look at yourself,” and then he hit me against that mirror. You know, sometimes he would just say he is doing me a favour staying married to me because in his heart, he knows he does not love me, and no wonder why sometimes he does not want to touch me. He said that there is someone that he loves, and he married me because he impregnated me at an early age.’ (P07, 32, employed)

The worth of the woman as a housewife was denied, and her natural physical beauty was criticised:

‘He tells me that I am useless and no one would ever love me. He says I’m not capable of doing anything – even my cooking is bad to him. I am dark in complexion and he would just say that he wants someone light in complexion, so he would buy me corticosteroids [*movate cream*] to apply so that I could be light, but I refuse because I feel so belittled, and I don’t want to be what I don’t like. I was “black” when he proposed and married me.’ (P09, 35, employed)

#### Sub-theme 1.2: Controlling behaviour of male partners

Participants mentioned that their husbands check their phones for calls and chats and control their movements.

A woman was not trusted and felt flattered about it:

‘His checking my cell phone and asking my whereabouts is part of marriage. This shows that he cares about me. I love his jealousy of my actions. Calling me names is not a big deal. I just say this is my man, he is just joking with me.’ (P08, 49, unemployed)

The woman experienced distress due to isolation; the reasons for her isolation was that the woman embarrassed the man and did not need to be in contact with her family:

‘I don’t attend our neighbours’ functions such as *tshisevhesevhe* [*a group of people who meet monthly to exchange gifts as friends*] because I am forbidden, and he said that I will humiliate him in front of people. This has ruined the relationship I had with people because they say I think I’m better than them and I am full of pride. I mean, I don’t even attend family gatherings back at home because he says I talk too much with my stinking mouth. He made me cut ties with my family and friends. There is nothing more painful than when your husband prevents you to see your mother.’ (P09, 35, employed)

#### Sub-theme 1.3: Withholding of affection and denial of conjugal rights

It was revealed during the interviews that some women were denied sex by their husbands.

Women are oppressed in that their feelings are not considered and they cannot initiate or be part of their love. They always go the way of the husband:

‘My husband is selfish and does not think for other people. I think he has a girlfriend, because most of the time he comes home late and does not want to engage in sexual activities. There are times when I will beg him just to touch me, even if he does not give me sex, but [*he*] would refuse. I am sure when he wants sex, he goes to his girlfriend.’ (P06, 38, employed)

A participant was hurt when she learned about her partner’s dishonesty:

‘It was very painful for me the time I discovered that he was cheating on me, because I used to ask myself what is it that I do not have that he wanted from other women. Maybe I don’t satisfy him [*laughs*]. Most of the time he comes back home late and would push me when I touch him, saying he is tired.’ (P03, 41, employed)

A participant wanted to get back at her husband by spiting him with inappropriate sexual behaviour, due to feeling hopeless:

‘The other time I thought of cheating because he does not spend weekends at home, whereas during the week, he uses work as a scapegoat, saying he needs time to rest. Unfortunately, who will propose to me? Because people around here are scared of him.’ (P05, 43, employed)

### Theme 2: Impacts of emotional abuse on the victim

Emotional abuse is the maltreatment of an individual in which a range of words are used intending to control, harm, undermine or terrify a person mentally and emotionally or influence a person’s thoughts and actions within their everyday lives. Sadness is viewed as a risk factor for a few mental and social problems. Research has demonstrated that an unpleasant life situation can be a critical indicator of sadness. Various unpleasant life-altering situations have been connected with sadness. A new report showed that openness to violence intellectually influences future convictions, increasing feelings of sadness. Also, having encountered rape is firmly connected with sadness (Parada-Fernández et al. 2021).

#### Sub-theme 2.1 Feeling of hopelessness and low self-confidence

Participants from the village shared their experiences. They felt that they have no value in their husbands’ lives because of the treatment they received. The study revealed that these women also experienced verbal abuse.

A participant had a lack of self-confidence in dressing and finding the purpose of her life, and she was unsure due to the suspicious behaviour on the husband’s phone:

‘Everything that he does to me makes me feel useless and worthless, and I feel that I do not have a purpose in life, and his words are painful. I just feel demotivated about doing anything positive in my life; it makes me look down on myself. When walking around, I think about what other people are saying about me being ugly, and I think that when they laugh, they are laughing at my appearance. I no longer have self-confidence. I started losing it the time he started laughing at the way I dress. I am usually stressed because of thinking about what he will do if I do this or that. I have lost trust in my husband because I believe that he is hiding something, and that is why he does not want me to check or answer his phone.’ (P02, 29, unemployed)

A participant needed sex and went so far as to partake in hurtful actions:

‘Begging for sex is the most humiliating thing a woman could do. He makes me feel unloved and cheap. Not having sex with someone you love is just depressing because I always ask myself where he is getting the sex. The day he would want sex, I would want us to have it in the dark because I am ashamed of my physique. I even try to do positions that are painful just to satisfy him so that he will not tease me but say something nice. But except for this, I feel that everything he does is out of love. What is love without being jealous? I previously thought of cheating on him because I am sexually frustrated.’ (P09, 35, employed)

#### Sub-theme: 2.2 Poor mental health

The married women in this village demonstrated poor mental health. It was revealed during the interviews that participants experienced anger, fear and withdrawal. The following quotes attest to this.

A participant thought she was not worthy to live and withdrew from interacting with people because of the treatment received from her husband:

‘You know, sometimes I think it is better to die because of the treatment that he gives me. Enough is enough, I heard it. Of late, I decided to remain indoors and avoid hearing horrible things out there; I am telling you, this makes me feel good.’ (P04, 48, employed)

The male partner’s comments left her with deep emotional scars, since nothing is more agonising than being informed that you are terrible by the individual who should safeguard you:

‘Sometimes I feel like I am outside my body and that I will wake up and find that it is a dream. I have scars within me because there is nothing more painful than being told that you are ugly by the person you love and who ought to protect you. My heart aches.’ (P07, 32, employed)

The unfaithfulness of the male partner made a participant feel embarrassed going outside, knowing that others would be snickering:

‘I am even ashamed of going out in the streets because they will be laughing at me due to his infidelity. I do not know if I am just being mistrustful or if it’s real.’ (P02, 29, unemployed)

Some married women expressed poor self-esteem, eating disorders, depression and suicidal thoughts:

‘Self-esteem is something that I lost a long time ago. I am always confused because one minute he loves me and then the next he does not. When he does not want to touch me, I will just be sitting there thinking about what could be the cause.’ (P07, 32, employed)

The pressure and sadness of knowing that the partner had extramarital affairs contributed to the development of an eating disorder:

‘I even developed a tendency of eating too much junk food as my way of escaping the pain from the abuse. I had two miscarriages in a year and a low-birth-weight baby last year due to the stress and depression I was facing at that time. I blame myself, because if my first-born son was his, maybe I wouldn’t be going through this. I even thought of killing myself for this mountain I am facing because he has a mistress.’ (P10, 37, employed)

The laughing of the partner without reason caused more pain to the participant and was attributed to a lack of decent shape:

‘It is difficult to please my husband. At times he would invite me to join him when attending events. I would choose my best dress to put on. Instead of saying encouraging words, he would laugh at me as if I were a fool. He would even tell me that I don’t know how to dress. This makes me feel bad.’ (P04, 48, employed)

### Theme 3: Reasons for remaining in an emotionally abusive marriage

Marriage comes from the Almighty God; it is indicated in the Bible that men are the heads of the families, and women are to submit themselves to their husbands. The participants perceived separation as unacceptable after pledging before God that only death would separate them. Furthermore, their experience of abuse may be informed by the cultural scripts by which they live. The following sub-themes emerged from the main theme.

#### Sub-theme 3.1: Commitment made before God

During questioning, participants revealed that they remained in abusive marriages for various reasons.

A participant perceived separating from a husband as unacceptable after pledging before God that only death would separate them:

‘It is a sin to divorce a man, according to the Bible, and I have made a commitment before God that I’m going to love this man through good and bad, meaning that I have to endure everything he throws at me. My silence is doing right by God, plus I am too old to be starting a new life. Marriage is forever.’ (P01, 63, unemployed)

#### Sub-theme 3.2: Poverty

The hope that a family has and neediness caused this participant to remain in the marriage:

‘Family expectations and poverty will make you stay. I also have a fear that my children will have problems starting a life in a new area with new people, and the fact of them changing schools is not a good idea because they might fail to adapt to the new environment and lose their self-confidence and fail.’ (P02, 29, unemployed)

#### Sub-theme 3.3: Love for the person

The love a participant had for her partner was another reason the participant remained in the marriage, irrespective of the abusive acts that the man committed against her:

‘I do not know if I could live without him, because he is my life. I love him so much, and whatever he does, I think it is out of love. A man must be jealous to show that he loves you. I do not have a choice but to stay, because he has done a lot for me and my family; I should also appreciate his efforts and not focus only on the bad things.’ (P06, 38, employed)

The participant was aware that she was emotionally abused, but continued to be in that relationship for fear of losing her partner:

‘He buys me presents after being emotionally abused as a way of apologising. The presents make me realise how much my husband loves me. As you know that there is a saying which says men are the same; even if I divorce him, the one I will get will do the same. There is no perfect man. It is better to have an emotionally abusive husband than to be single. What I like about him is that he does not assault me. As you can see, I don’t have scars. People will think I am mad if I leave my caring husband. Cheating should not be the reason for a woman to leave her husband, as all men cheat.’ (P03, 41, employed)

## Discussion

The findings of the study revealed that married women in the selected village are emotionally abused within their homes by their husbands in a similar manner daily. Participants have different understandings of emotional abuse. Some women do not consider emotional abuse as abuse or something serious; abuse, to them, must be physical. Some see it as something normal, and others see it as private and believe it should be a family secret. Moono et al. (2020) and Smith (2019) related secrecy to social standing within the community, as the family would be shamed should the abuse be divulged to other people. Furthermore, women’s reluctance to disclose their experiences of abuse may be informed by the cultural scripts by which they live.

Women are treated as subjects that must put up with all types of behaviour from their husbands. They are demeaned, shamed, belittled and made to feel inferior because they believed that they should always be submissive and obedient to their husband and their loyalty towards them. The cultural institutions that support *lobola*, which disempowers women and reinforces violence and acceptance of violence, gave participants’ husbands power and control over their wives. The participants thought men had every right to do whatever they liked with them because they had paid their bridal price (*lobola*). This is in line with Moono et al. (2020) and Muche et al. (2017), who revealed that *lobola* is a cultural practice that leads to gender violence and abuse of cultural norms and practices, which could be the case in this study.

Participants in this study were controlled by their partners. Some believed that a husband can tell the wife what to wear, whom to talk to and how to behave in public. They did not see anything wrong with that. For them, being prevented from going somewhere and accused of infidelity was a way of showing love. The participants believed that when men constantly monitored their cell phones, it showed love, not abuse, even though they were not allowed to monitor their husbands’ phones. He invaded her privacy without her invading his. This is in line with Legg and Pietrangelo (2018) and Ogland et al. (2014) who found that women perceive jealousy as part of being loved.

Women reported begging for sexual activities and felt they were not good enough. Some stated that their partners withdrew sex, which made them sexually frustrated and lonely. Sexual desire depends on emotions and feeling relatively free of criticism from the partner. A woman must feel safe and relaxed when engaging in sexual activities and not be ridiculed in the process, because a woman might feel dirty and used after sexual intercourse. Sexual problems like sexual withdrawal cause marital dissatisfaction, as this cannot be discussed with anyone for fear of embarrassment. Bonilla-Algovia, Rivas-Rivero and Vazquez (2020) concurred that emotional abuse destroys marriages because the woman is dying inside without talking to someone about the problem.

Emotional abuse can lead to poor mental health such as depression, anxiety and many more symptoms. It is not surprising that participants revealed symptoms of poor mental health such as stress, social withdrawal, poor sleep and being unable to think properly. The experiences could even escalate to the thought of ending one’s own life, as indicated by the participants in the current study, if not addressed. A study by Martins-Monteverde et al. (2019) demonstrated that any embarrassing behaviour could lead to depression. This could mean the experience of emotional abuse due to their husbands’ infidelity or the demeaning experience of sex being withdrawn by their husbands can cause depression in participants, which may lead to suicide.

The participants remained in their marriages after experiencing the abuse for various reasons. Obedience to God made emotionally abused women remain in the marriage and be submissive to their husbands. The finding is in line with Ersoy and Yildiz (2011), who revealed that married women remained in abusive marriages because they were afraid that the violence could escalate once they ended the marriage. Some participants thought it was the right thing to do for their children because they did not want them to lose their identity, and starting a new life in a new environment could be devastating, and they might fail to adapt. Participants tolerated violence and maintained the marriage because of economic dependence, especially the unemployed, as they were dependent on their husbands for financial assistance; should they leave, they would have no financial assistance for themselves and their children (Moono et al. 2020).

Domestic violence is often seen through a stereotypical lens in which battered women endure mental damage from their partners and continue to remain in the relationship (Brosi et al. 2020). The finding of this study concurs with previous studies that women stay with their harmful abusive partners because of affection. Women may remain on account of the adoration they have for their partners (Heron, Eisma & Browne 2022; Sichimba, Nakazwe & Phiri 2020). Frequently, the pattern of abuse makes it challenging for women to break free, and psychologists ought to keep on giving support regardless of the woman’s readiness to leave a harmful relationship (Lutgendorf 2019).

### Limitations of the study

The study was conducted among women in one village in the Limpopo province, and the findings cannot be generalised to all women in the province.

## Conclusion

The study increased understanding of the experiences and consequences of emotional abuse among married women. The study shows a lack of understanding and consequences of emotional abuse among married women in a village in Limpopo province. Some saw it as something normal, and others saw it as private and something that should be a family secret. Women were demeaned and made to feel inferior and were dying inside because they could not discuss sexual withdrawal with anyone for fear of embarrassment. The cultural institutions that support *lobola* disempower women and reinforce violence, allowing husbands to have power over their wives. The participants thought men had every right to do whatever they liked with them because they had paid their bridal price (*lobola*).

Hence, it is essential to increase the understanding of emotional abuse among women by educating them on various platforms. Awareness campaigns may improve the perception of those who do not view emotionally abusive actions as abuse, particularly those with low educational levels and the illiterate. Psychologists should counsel married women experiencing emotional abuse at home to enable them to make informed decisions about their future. Support groups may mitigate the symptoms of poor mental health expressed by these women.

## References

[CIT0001] Abrahams, N., Mathews, S., Lombard, C., Martin, L.J. & Jewkes, R., 2017, ‘Sexual homicides in South Africa: A national cross-sectional epidemiological study of adult women and children’, *PLoS One* 12(10), e0186432 29040329 10.1371/journal.pone.0186432 PMC5645125

[CIT0002] Auteri, S., 2019, *Women are having unwanted sex to maintain their relationships*, viewed 30 October 2021, from https://www.instyle.com.

[CIT0003] Bonilla-Algovia, E., Rivas-Rivero, E. & Vazquez, J.J., 2020, ‘Impact of gender-based violence on psychological distress and happiness in Leon (Nicaragua)’, *Health Care for Women International* 41(6), 673–689. 10.1080/07399332.2020.1764564 32420818

[CIT0004] Bradbury-Jones, C., & Isham, L., 2020, ‘The pandemic paradox: The consequences of COVID-19 on domestic violence’, *Journal of Clinical Nursing* 29(13–14), 2047–2049. 10.1111/jocn.15296 32281158 PMC7262164

[CIT0005] Brosi, M., Rolling, E., Gaffney, C. & Kitch, B., 2020, ‘Beyond resilience: Glimpses into women’s posttraumatic growth after experiencing intimate partner violence’, *The American Journal of Family Therapy* 48(1), 1–15. 10.1080/01926187.2019.1691084

[CIT0006] Christian Aid Ireland Learning Paper, 2018, *Gender-based violence programming in contexts affected by violence and conflict*, viewed 15 November 2021, from https://tec.alnap.org/.

[CIT0007] Creswell, J., 2013, *Research design qualitative, quantitative and mixed methods approaches*, Sage Publications Inc, Thousand Oaks, CA.

[CIT0008] Creswell, J.N. & Poth, N.P., 2018, *Qualitative inquiry and research design: Choosing among five approaches*, Sage Publications Inc, London.

[CIT0009] Denzin, N.K. & Lincoln, Y.S., 2011, *The SAGE handbook of qualitative research*, Sage Publications Inc, Thousand Oaks, CY.

[CIT0010] De Vos, A.S., Strydom, H., Fouche, C.B. & Delport, C.S.L., 2011, *Research at grassroots for the social sciences and human services professions*, Van Schaik, Pretoria.

[CIT0011] Ersoy, O.Ç. & Yildiz, H., 2011, ‘Reproductive health problems and depression levels of women living in sanctuary houses as a result of husband violence’, *Health Care for Women International* 32(9), 795–810. 10.1080/07399332.2011.565528 21834719

[CIT0012] Field, S., Onah, M., Van Heyningen, T. & Honikman, S., 2018, ‘Domestic and intimate partner violence among pregnant women in a low resource setting in South Africa: A facility-based, mixed methods study’, *BMC Women’s Health* 18, 119. 10.1186/s12905-018-0612-2 29973182 PMC6030741

[CIT0013] CoGTA.gov.za, 2020, *Vhembe District Municipality: Profile and analysis*, viewed 14 November 2021, from https://www.cogta.gov.za.

[CIT0014] Grove, S. & Gray, J., 2018, *Understanding nursing research: Building an evidence-based practice*, Elsevier, Saunders, TX.

[CIT0015] Grove, A.K., Moodley, D., McNaughton-Reyes, L., Martin, S.L., Foshee, V. & Maman, S., 2015, ‘Prevalence, rates and correlates of intimate partner violence among South African women during pregnancy and the postpartum period’, *Maternal and Child Health Journal* 19(3), 487–495. 10.1007/s10995-014-1528-6 24889116 PMC4254902

[CIT0016] Heron, R.L., Eisma, M. & Browne, K., 2022, ‘Why do female domestic violence victims remain in or leave abusive relationships?’, A qualitative study’, *Journal of Aggression, Maltreatment & Trauma* 31(5), 677–694.

[CIT0017] Heydarikhayat, N., Ghanbarzehi, N., Shahkaramzehi, Z., Sabagh, K. & Rohani, C., 2022, ‘Nurses’ lived experiences of caring for patients with COVID-19: A phenomenological study’, *Journal of Research in Nursing: JRN* 27(4), 313–327. 10.1177/17449871221079175 35837262 PMC9272502

[CIT0018] Kimuna, S., Tenkorang, E.Y. & Djamba, Y., 2018, ‘Ethnicity and intimate partner violence in Kenya’, *Journal of Family Issues* 39(11), 2958–2981. https://doi.org/10.1177%2F0192513X18766192

[CIT0019] Legg, T.J. & Pietrangelo, A., 2018, ‘How to recognize the signs of mental and emotional abuse’, *Healthline*, viewed 10 November 2021, from https://www.healthline.com/health/signs-of-mental-abuse.

[CIT0020] Li, E.T., Carracher, E. & Bird, T., 2020, ‘Linking childhood emotional abuse and adult depressive symptoms: The role of mentalizing incapacity’, *Child Abuse and Ne glect* 99, 104253. 10.1016/j.chiabu.2019.104253 31812024

[CIT0021] Lutgendorf, M.A., 2019, ‘Intimate partner violence and women’s health’, *Obstetrics & Gynecology* 134(3), 470–480. 31403968 10.1097/AOG.0000000000003326

[CIT0022] Martins-Monteverde, C.M.S., Baes, C.V.W., Reisdorfer, E., Padovan, T., Tofoli, S.M.D.C. & Juruena, M.F., 2019, ‘Relationship between depression and subtypes of early life stress in adult psychiatric patients’, *Frontiers in Psychiatry* 10, 19. 10.3389/fpsyt.2019.00019 30804815 PMC6370718

[CIT0023] Moono, P., Thankian, K., Menon, G.B., Mwaba, S.O.C. & Menon, J.A., 2020, ‘Bride Price (Lobola) and Gender-based Violence among Married Women in Lusaka’, *Journal of Education, Society and Behavioural Science* 33(8), 38–47; Article no.JESBS.60758.

[CIT0024] Mittal, S. & Singh, T., 2020, ‘Gender-based violence during COVID-19 pandemic: A mini-review’, *Women’s Health* 1, 4. 10.3389/fgwh.2020.00004 PMC8594031 34816149

[CIT0025] Morrow, R., Rodriguez, A. & King, N., 2015, ‘Colaizzi’s descriptive phenomenological method’, *The Psychologist* 28(8), 643–644.

[CIT0026] Mshweshwe, L., 2020, ‘Understanding domestic violence: Masculinity, culture, traditions’, *Heliyon* 6(10), e05334. 33150213 10.1016/j.heliyon.2020.e05334 PMC7599123

[CIT0027] Muche, A.A., Adekunle, A.O. & Arowojolu, A.O., 2017, ‘Gender-based violence among married women in Debre Tabor Town, Northwest Ethiopia: A qualitative study’, *African Journal of Reproductive Health* 21(4), 102–109. 10.29063/ajrh2017/v21i4.11 29624956

[CIT0028] Muluneh, M.D., Stulz, V., Francis, L. & Agho, K., 2020, ‘Gender-based violence against women in sub-Saharan Africa: A systematic review and meta-analysis of cross-sectional studies’, *International Journal of Environmental Research and Public Health* 17(3), 903. 10.3390/ijerph17030903 32024080 PMC7037605

[CIT0029] Neubauer, B.E., Witkop, C.T. & Varpio, L., 2019, ‘How phenomenology can help us learn from the experiences of others’, *Perspectives on Medical Education* 8, 90–97. 10.1007/s40037-019-0509-2 30953335 PMC6468135

[CIT0030] Ogland, E.G., Xu, X., Bartkowski, J.P. & Ogland, C.P., 2014, ‘Intimate partner violence against married women in Uganda’, *Journal of Family Violence* 29(8), 869–879. 10.1007/s10896-014-9640-3

[CIT0031] Okolie, E.Q., 2019, ‘Critical analysis of state of women’s rights in Nigeria’, *International Journal of African and Asian Studies*. 10.7176/JAAS/53-07

[CIT0032] Parada-Fernández, P., Herrero-Fernández, D., Oliva-Macias, M. & Rohwer, H., 2021, ‘Stressful life events and hopelessness in adults: The mediating role of mentalization and emotional dysregulation’, *Brazilian Journal of Psychiatry* 43(4), 385–392. 10.1590/1516-4446-2020-1061 33084731 PMC8352732

[CIT0033] Perrin, N., Marsh, M., Clough, A., Desgroppes, A., Phanuel, C.Y., Abdi, A. et al., 2019, ‘Social norms and beliefs about gender-based violence scale: A measure for use with gender-based violence prevention programs in low-resource and humanitarian settings’, *Conflict and Health* 13, 6. 10.1186/s13031-019-0189-x 30899324 PMC6408811

[CIT0034] Sapkota, D., Bhattarai, S., Baral, D. & Pokharel, P.K., 2016, ‘Domestic violence and its associated factors among married women of a village development committee of rural Nepal’, *BMC Research Notes* 9, 178. 26994899 10.1186/s13104-016-1986-6 PMC4799562

[CIT0035] Saunders, B., Sim, J., Kingstone, T., Baker, S., Waterfield, J., Bartlam, B. et al., 2018, ‘Saturation in qualitative research: Exploring its conceptualization and operationalization’, *Quality & Quantity* 52(4), 1893–1907. 10.1007/s11135-017-1574-8 29937585 PMC5993836

[CIT0036] Smith, K., 2019, *Warning signs of an abusive husband*, viewed 21 December 2021, from https://www.guystuffcounseling.com.

[CIT0037] Smith, K.I., 2020, *Counting dead women*, viewed 18 December 2021, from https://kareningalasmith.com/.

[CIT0038] Sichimba, F., Nakazwe, K.C. & Phiri, T., 2020, ‘Untold stories of women living in violence: Lived realities of why women stay: A case study of Ngombe and Kanyama compounds in Lusaka’, *Journal of Aggression, Maltreatment & Trauma* 29(7), 767–784.

[CIT0039] South African Government, 2019, *National Gender-based Violence & Femicide Strategic Plan 2020–2030*, viewed 28 December 2021, from https://www.gov.za.

[CIT0040] South African Government, 2021a, *President Ramaphosa’s speech*, viewed 30 December 2021, from https://www.gov.za/speeches/president-cyril-ramaphosa-virtual-fund-raising-event-private-sector-gender-based-violence.

[CIT0041] South African Government, 2021b, *Violence against women and children*, viewed 30 December 2021, https://www.gov.za/issues/violence-against-women-and-children-0#.

[CIT0042] The World Bank, 2019, *Gender-based violence (Violence against women and girls)*, viewed 20 November 2021, from https://www.worldbank.org.

[CIT0043] Tracy, N., 2021, ‘Emotional abuse: Definitions, signs, symptoms, examples’, *HealthyPlace*, viewed 04 January 2022, from https://www.healthyplace.com.

[CIT0044] Tsai, A.C., Tomlinson, M., Comulada, W.S. & Rotheram-Borus, M.J., 2016, ‘Intimate partner violence and depression symptom severity among South African women during pregnancy and postpartum: A population-based prospective cohort study’, *PLoS Medicine* 13(1), e1001943. 10.1371/journal.pmed.1001943 26784110 PMC4718639

[CIT0045] Vhembe.gov.za, 2021, *The Vhembe District Municipality*, viewed n.d., http://vhembe.gov.za/district/location.

[CIT0046] Wanger, S., 2020, ‘Domestic violence growing in wake of coronavirus outbreak’, *The Conversation*, viewed 29 October 2021, from https://theconversation.com.

